# Development of comparable algorithms to measure primary care indicators using administrative health data across three Canadian provinces

**DOI:** 10.23889/ijpds.v5i1.1340

**Published:** 2020-08-11

**Authors:** MW Alsabbagh, JK Kueper, ST Wong, F Burge, S Johnston, S Peterson, B Lawson, H Chung, M Bennett, S Blackman, K McGrail, J Campbell, W Hogg, R Glazier

**Affiliations:** 1 University of Waterloo; 2 The University of Western Ontario; 3 University of British Columbia; 4 Dalhousie University; 5 Bruyère Research Institute, University of Ottawa; 6 ICES; 7 University of Exeter; 8 University of Ottawa, Montfort Hospital Research Institute

## Abstract

**Introduction:**

Performance measurement has been recognized as key to transforming primary care (PC). Yet, performance reporting in PC lags behind even though high-performing PC is foundational to an effective and efficient health care system.

**Objectives:**

We used administrative data from three Canadian provinces, British Columbia, Ontario and Nova Scotia, to: 1) identify and develop a core set of PC performance indicators using administrative data and 2) examine their ability to capture PC performance.

**Methods:**

Administrative data used included Physician Billings, Discharge Abstract Database, the National Ambulatory Care and Reporting System database, Census and Vital Statistics. Indicators were compiled based on a literature review of PC indicators previously developed with administrative data available in Canada (n=158). We engaged in iterative discussions to assess data conformity, completeness, and plausibility of results in all jurisdictions. Challenges to creating comparable algorithms were examined through content analysis and research team discussions, which included clinicians, analysts, and health services researchers familiar with PC.

**Results:**

Our final list included 21 PC performance indicators pertaining to 1) technical care (n=4), 2) continuity of care (n=6), and 3) health services utilization (n=11). Establishing comparable algorithms across provinces was possible though time intensive. A major challenge was inconsistent data elements. Ease of data access, and a deep understanding of the data and practice context, was essential for selecting the most appropriate data elements.

**Conclusions:**

This project is unique in creating algorithms to measure PC performance across provinces. It was essential to balance internal validity of the indicators within a province and external validity across provinces. The intuitive desire of having the exact same coding across provinces was infeasible due to lack of standardized PC data. Rather, a context-tailored definition was developed for each jurisdiction. This work serves as an example for developing comparable PC performance indicators across different provincial/territorial jurisdictions.

## Introduction

Reporting on performance can influence quality improvement agendas and improve performance [1]. Past work shows that public reporting may improve performance,[2–6] as it has the potential to “improve the quality of care, increase accountability, facilitate public participation in health care,” [7–9] impact societal and professional values, and direct attention to issues not currently on the policy agenda [7,10,11]. It may also facilitate collaboration among stakeholders as they set a common agenda [12]. Performance reporting in the hospital sector continues to grow. Yet, performance reporting in primary care (PC) lags behind even though high-performing PC is widely recognized as foundational to an effective and efficient health care system. Countries with strong PC systems have lower mortality rates and overall costs as well as better health outcomes and health equity [13–15].

There are growing demands for performance reporting in PC from many stakeholders including patients [16,17]. Performance measurement has been recognized as key to transforming PC [18–20]. This includes comparison of performance against both internal and external standards, and identifying opportunities for improvement [21]. However, PC performance reporting is challenging because of the dearth of concise and synthesized information, and because many clinicians prefer to be accountable only for their individual role and do not view themselves as actors within a larger system [22].

There are examples of national public reporting of PC performance in other countries but only limited efforts in Canada. International examples include BEACH in Australia [5,23], National Ambulatory Medical Care Survey (NAMCS) in the USA [18] and the Quality and Outcomes Framework in the UK [24,25]. There has been some provincial PC reporting by provincial Health Quality Councils [26,27]. The only significant national effort in Canada was the joint Canadian Institute for Health Information (CIHI)/Health Council report of a 2008 population survey [19]. The most commonly referenced performance information about PC in Canada is from the Commonwealth Fund patient and clinician surveys in industrialized nations [2,28–32]. The surveys are based on samples of 1000 patients or clinicians per country in independent surveys, and show that PC performance in Canada is poor compared to other countries. These disappointing results have helped put Community-Based Primary Health Care on Canada’s policy radar. Yet, the Commonwealth Fund surveys have limitations. Notably the small sample size does not permit meaningful analysis at the regional level where policy decisions are often made.

The creation of comparable information from PC data across jurisdictions remains nascent. Comparable information within and across jurisdictions is needed to ultimately influence health and healthcare outcomes. Moreover, this task is relevant for all learning healthcare systems,[33] particularly in federated jurisdictions like Canada, where there are multiple parallel provincial and territorial single-payer systems [34]. Because the fundamentals and objectives of PC are universal,[35] the transfer of learning experiences from one jurisdiction to another should be facilitated, especially within one country. This undertaking includes creating and maintaining high quality data as well as work towards gaining meaning from existing healthcare data within integrated healthcare systems.

Measuring PC requires multiple sources of information, including data from patients, clinicians, charts and administrative data [36]. While comprehensive PC information and measurement systems are still being built, one obvious place to start is using health administrative data since it is already routinely collected. These data are longitudinal and can provide actionable information about healthcare services [37–39]. For example, Health Quality Ontario uses administrative data to provide PC clinicians with reports on performance of care and health service utilization particular to their practices’ patient panels [40]. The purpose of this work was to 1) identify and develop a core set of indicators of PC performance using administrative health data and 2) examine their ability to capture PC performance. This paper reports on the processes of doing this work, including the infrastructure and resources needed to support it, noting challenges and examples of how to promote similarity when working with administrative data from multiple jurisdictions. While this work relied only on Canadian data from three separately funded and managed provincial health systems, it holds lessons for other efforts to compare data across health systems.

## Methods

This work took place as part of the TRANSFORMATION study, which set out to improve the science and reporting of PC performance. TRANSFORMATION is a cross-sectional, multi-site research program [41] in Canada that used multiple sources of data (patient, provider and organizational surveys, administrative data, case studies, and deliberative dialogues with patients and clinicians) to produce comprehensive regional-level PC performance reports. The study sites, Fraser East, British Columbia (BC), Eastern Ontario Health Unit, Ontario (ON), and Central Zone, Nova Scotia (NS) based on the willingness of the clinicians and decision-makers in these areas to participate. Herein, we describe our methodology for developing PC reporting using the administrative data.

*Administrative Data source*. Canada’s 13 provincial/territorial (P/T) governments are responsible for delivery and organization of healthcare where the government is the insurer and administers its own version of a healthcare plan [21]. Healthcare is paid for through federal government transfers and P/T public tax revenues [42]. In terms of PC, most of it is delivered through family physicians who work in private practices, essentially small businesses [43]. These businesses generate data because they bill for services. The single largest source of PC administrative data is provincial billing data. In addition, specific provincial data sources were accessed and used for this study relating to health coverage registration and services delivery (Table 1).

**Table 1: Databases used in the TRANSFORMATION Study:** Abbreviations: DAD: Discharge Abstract Database, OMHRS: Ontario Mental Health Reporting Systems, MED: MSI Physician’s Billings, MSP: Medical Services Plan, NACRS: National Ambulatory Care Reporting System, ODB: Ontario Drug Benefit Claims, OHIP: Ontario Health Insurance Plan, RPDB: Registered Persons Database

	British Columbia	Ontario	Nova Scotia

Common datasets			
Acute hospital discharges [45]	✓	✓	✓
	DAD	DAD	DAD
Mental Health hospital discharges	✓	✓	✓
	DAD	OMHRS (admissions to psychiatric hospitals or acute hospitals with psychiatric beds) and DAD (mental health admissions to acute care hospitals without psychiatric beds)	DAD
Day surgeries	✓	✓	✓
	DAD	NACRS	NACRS
Emergency department (ED) visits. Data coverage and details depend on the province [44].	✓	✓	✓
	The combination of 2 data sources, NACRS [44] and MSP [46] (contains the majority of ED visits). DAD was used for ED visits that result in hospital admission.	NACRS	The combination of 2 data sources, NACRS and MED (contains the majority of ED visits). DAD was used for ED visits that result in hospital admission.
Physician billings	✓	✓	✓
Data coverage and details depend on the province.	MSP	OHIP	MED
PharmaCare	✓	✓	✓
Data coverage and details depend on the province.	PharmaNet [47]	ODB	Senior’s Pharmacare (PHARM)
Physician database	✓	✓	✓
	MSP Practitioner database [48]	ICES Physician Database	Licensed Provider Registry (DOCTORS)
Registry information	✓	✓	✓
	Consolidation File (MSP Registration & Premium Billing) [46]	RPDB	Master Insured Patient Registry
Citizenship and Immigration	Study period years not available.	✓	✓
		Permanent Residents IRCC Permanent Residents database	Master Insured Patient Registry
Vital statistics	✓	Study period years not available.	✓
	Vital Statistics- Deaths [49]		Vital Statistics - Death
Other databases	-Census data [50]	-Ontario Census Area Profiles	-Patient Geography
		-Client Profile Database	
		-Corporate Provider Database	
		-OHIP’s Emergency Claims Database	
		-Ontario Cancer	

Several administrative datasets were common across all three provinces (Table 1), including the Discharge Abstract Database (DAD) and — to a lesser degree — the National Ambulatory Care and Reporting System Metadata (NACRS) database [44,45]. While DAD coverage and data elements were consistent across all provinces, differences in NACRS were present. In ON, detailed data pertaining to emergency department visits and other outpatient services (such as day surgeries) from all facilities were collected. For the other two provinces, emergency department visits were only in NACRS from some facilities; these data were combined with physician billings to capture as many emergency department visits as possible. Further, while day surgery was available in NACRS for NS, it was only available in DAD for BC.

All jurisdictional data can be linked within province using a unique identifier. That is, the linkage of datasets can be completed by the data hosting agency and the research team can access a de-identified linked dataset. However, these linked jurisdictional data can only be analyzed within each province. Population Data BC [51], ICES (formerly the Institute for Clinical Evaluative Sciences) in ON [52], and Health Data NS [53] provide their province’s secure research environment that is used for analysis. Each organization provides access to administrative data for healthcare research. Details about the work environment for data access in each province is available in Supplementary Appendix 1.

Although these organizations have similar purposes to protect patient confidentiality, significant differences in terms of experience and available resources are also observed among them [51]. Specifically, ICES has considerable funding and capacity for work with administrative databases not available to the same extent in BC or NS. These differences led to additional complexities in our ability to analyze the data.

*PC performance indicators*. Indicators of PC performance were compiled based on a literature review of PC indicators previously developed with administrative data available in Canada (n=158),[1,3–7,9,10,52] including reviewing the previously developed CIHI indicators [53–62] (see supplemental material: S_Table 1). For each indicator, we ascertained if the requisite administrative data were available and whether there was a pre-existing algorithm being used to measure a similar construct or whether the indicator needed to be developed for this study specifically. The research team shortened the list using the following inclusion criteria: 1) indicator measures PC performance and 2) data used to construct the indicator were available in at least two out of the three provinces — this was reduced from three due to so few indicators being possible to compute in all three provinces. To the extent possible, we constructed the cross-provincial list of indicators from each province’s data elements so that they would produce comparable algorithms to measure of PC performance.

*Mapping PC performance indictors to a theoretical framework*. In order to ensure that identified indicators target key theoretical aspects of PC performance and to assess which of these theoretical domains are possible to address with administrative data [63], they were mapped to the Hogg et al. (2008) framework [37]. This PC Performance Measurement Framework, which includes core PC performance domains, was chosen as it provides a comprehensive view of PC performance [36,37,64,65]. Indicators fitting into this framework demonstrates a high potential to help improve patient care.

*Analysis to assess algorithm comparability of PC indicator*. Knowlton et al.’s (2017) framework pertaining to aligning data from multiple sources was used to assess the province’s algorithms used to create the performance indicators [66]. Specifications of each candidate PC indicator was defined. We engaged in iterative discussions to assess data conformity, completeness, and plausibility of the results pertaining to PC performance indicators in all jurisdictions. We maintained a file of detailed documentation and a spreadsheet of the decisions, a process known as ‘data curation’ [67].

The documentation was used to record the process of establishing the comparable algorithms for PC performance. The documentation included definitions of what was recorded in the datasets, inclusion and exclusion criteria such as age and sex restriction to create a cohort for each indicator, name(s) and type(s) of the variables used in the denominator and numerator of performance indicator calculations, and any additional information needed to understand each indicator. Notes and comments also documented whether indicators are routinely computed for ongoing performance measurement, such as indicators in the ICES Primary Care Population cohort (PCPOP) that are used for Ontario’s performance reports to family physicians or if indicators required new or adapted algorithms.

Comparable algorithms were assessed by examining the data source and data elements used to construct the indicator in each province, any modifications made to the original measurement, ease of adaptability for each province to achieve a final version of the indicator, and the practice context within each province. Challenges in examining and creating comparable algorithms was examined through content analysis of the notes and working with the research team, which included clinicians, analysts, and health services researchers familiar with PC.

## Results

**Figure 1: Flow diagram of indicator selection process:**
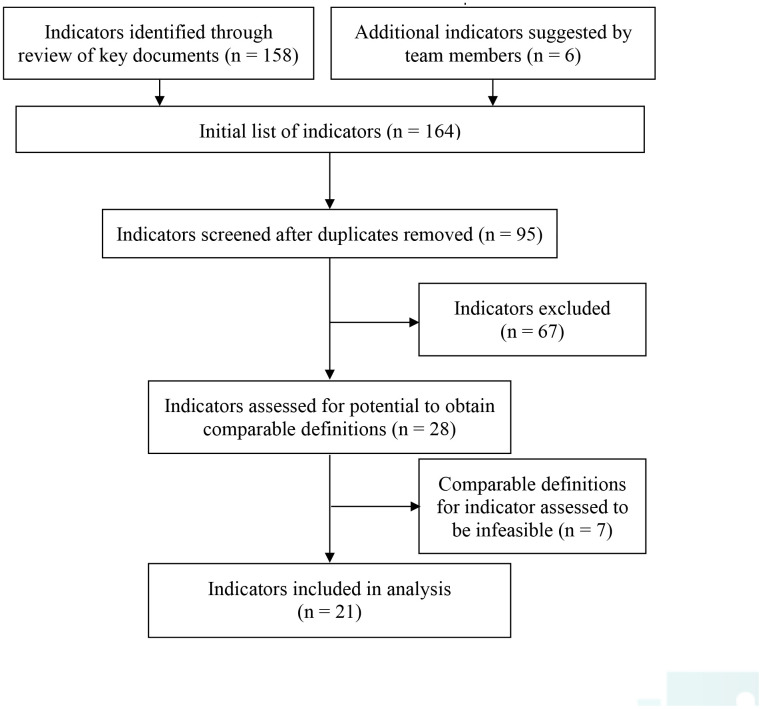


*PC performance indicators*. We initially assessed a total of 168 potential indicators. There were 158 indicators identified through the literature review (see supplementary material). After removing duplicates (n=69), an additional 67 indicators were removed because they did not meet inclusion criteria. An additional seven indicators were removed because their construction would either require excessive time to create a suitable version of the indicator or a comparable definition was not possible to operationalize across data sources available in the provinces (Figure 1). For example, colon cancer screening occurs differently across the three provinces. In BC it is performed within PC; in NS, it is a government run service outside of PC; and in ON, it is a government organized program, where PC is incentivised and encouraged to perform, and the results are centrally monitored. Accordingly, colon cancer screening would not be an appropriate PC performance indicator in NS and ON, as it is not performed by PC in NS and is organized by the government in ON.

A total of 21 indicators were included in our study (Table 2). These indicators can be categorized as: 1) technical care (n=4; cervical cancer and osteoporosis screening, diabetes management, and use of metformin; 2) continuity of care (n=6; continuity of care index, usual provider of care index, modified continuity index, continuity with family physicians, mental health continuity, and multiple conditions continuity); and 3) health services utilization (n=11; emergency department visits, hospital readmissions, serious diabetes complication, mental health readmission, home visits in end-of-life, ambulatory care sensitive admissions, primary care costs per patient, total costs per patient, physicians prescribing medications per patient, number of different medications, and total medication costs per patient aged 65 and older) as per the Hogg et al framework [68].

**Table 2: Administrative data primary care indicators:** 

Primary Care Performance Indicator	Availability across provinces: All three provinces (BC, ON, NS) or only two out of three

Technical Quality of Care
Cervical cancer screening: Percentage of screening eligible patients up-to-date with Papanicolaou (Pap) tests	NS and ON only
Osteoporosis screening: Percentage women who turn 65 in fiscal year 2013-14, who had a bone mineral density test in the 3-year study period	All three
Diabetes management incentive code: Diabetes Management Incentive Code billed at least once in the most recent fiscal year of data	BC and ON only
Metformin: Proportion of patients diagnosed with diabetes mellitus who were prescribed Metformin as their first hypoglycemic agent	All three
Continuity of Care
	
Continuity of Care Index (COCI) to primary care providers: The COCI weights both the frequency of ambulatory visits to each primary care provider and the dispersion of visits among providers. The COCI values range from 0 (each visit made to a different primary care provider) to 1 (all visits made to a single primary care provider)	All three
Usual Provider of Care (UPC) Index: The proportion of ambulatory visits to the family physician (primary care provider) to whom they are attributed (virtually rostered) relative to all ambulatory visits to primary care providers (GPs) in the 3-year observation period	All three
Modified Modified Continuity Index (MMCI) [1 - number of different Primary Care providers seen ⁄ (number of contacts with a Primary Care providers + 0.1)] /
(1 - 1⁄(number of contacts with a Primary Care providers +0.1))	All three
Continuity with family physicians: Percentage of patients who saw fewer than five individual family physicians for ambulatory care in 1 fiscal year	All three
Mental health continuity: *(a) Any follow-up within seven days:* Percentage of patients hospitalized for a mental health reason who had a follow-up office visit within seven days after discharge
(b) *Shared care within 30 days:* Percentage of patients hospitalized for a mental health reason who had at least one follow-up office visit with a comprehensive primary care physician and at least one follow-up visit with a psychiatrist within 30 days after discharge	All three
Multiple conditions continuity: *(a) Any follow-up within seven days:* Percentage of patients hospitalized for COPD, diabetes, asthma, pneumonia or unstable angina who had an office visit within seven days after discharge
*(b)* Shared care within 30 days: Percentage of patients hospitalized for COPD, diabetes, asthma, pneumonia or unstable angina who had shared care within 30 days after discharge	All three

Technical Quality of Care

Emergency department (ED) visits:
(a) Total ED visits: rate per 1000
(b) Percent of ED visits that were urgent (CTAS 1-3) and less urgent (CTAS 4-5) among visits that has CTAS available
(c) ED visits that result in admission to hospital	(a) & (c): All three (b) All three, but for BC & NS it is based on a subset of ED visits
Hospital readmissions:
(a) Within 30 days
(b) Within 1 year	All three
Serious diabetes complication:
Percentage of people with diabetes who had a serious complication of diabetes (amputation, hospitalization for certain chronic conditions, or death), annualized	All three
Mental health readmission:
Percentage of patients hospitalized for mental health reasons who were readmitted for any reason within 30 days after discharge	All three
Home visits in end-of-life:
Proportion of people who received home visits from a family physician in last 3 months of life, whose cause of death was:
(a) Cancer
(b) Potentially advanced chronic disease (all other causes excluding cancer, obstetrics-related, accidents, & self-harm)	BC and NS
Ambulatory care sensitive admissions:
Number of non-elective hospital admissions for each of asthma, COPD, diabetes, and CHF, among people diagnosed with those conditions. In each cohort, rates were per 1,000 people.	All three
Primary care costs per patient: Mean ambulatory primary care costs per capita over a 1-year observation period	All three
Total costs per patient: Mean total healthcare costs (excluding prescription medication) per capita over a 1-year observation period and mean total healthcare costs (including prescription medication) per patient aged 65+ over a 1-year observation period.	All three
Physicians Prescribing per patient: Mean number of unique GPs prescribing medications per capita aged 65+, in a one-year period.	All three
Number of different medications:
Mean # of different medications per capita (from all prescribers, and, separately, only from GP prescribers) aged 65+, as measured at the ATC 4th level (chemical/therapeutic/pharmacological subgroup)	All three
Total Medication Costs per capita aged 65+:
Mean (and median) total Government prescription medication costs per capita (from all prescribers, and, separately, only from GP prescribers) aged 65+ over a 1-year observation period.	All three

*Comparable algorithms of PC performance*. Eighteen out of the 21 indicators could be constructed in all three provinces (Table 2). Comparable algorithms were easiest to achieve when common datasets across provinces were used (i.e. prepared according to a common set of standards as set by CIHI). The analysts in each jurisdiction ensured that the data could be maximized into comparable formats.

*Challenges in establishing comparable algorithms*. A major challenge in establishing comparable algorithms was the significant differences in the data elements. Ease of access to the data, in addition to a deep understanding of the data and practice context was essential to decide upon the most appropriate data elements within the data sources. Ontario’s critical mass of analytic and clinical capacity developed over a number of years versus BC and NS “younger” abilities to use these data added to the complexity of cross-jurisdictional work in PC. These single-center structures have heterogeneous data access request requirements, varying funding arrangements for internal analytic capacity and mission statements that drive the ability to create and refine a library of data programs. For example, ON researchers can conduct multiple analyses for a single dataset, although privacy and organizational approval must be attained and dataset requirements followed for each analysis. Researchers in BC and NS can only conduct analyses that have been approved, while also requiring an amendment or new data access request. Moreover, sustained provincial government funding and building analytic capacity within ICES has meant ON has dedicated significantly more time and resources fine-tuning their specialized databases (e.g. Ontario Diabetes Database) and algorithms (e.g. series of codes used to identify cancer screening indicators) to increase precision of indicator estimates.

It is current practice for ICES to publicly report on PC performance indicators whereas this investment in healthcare system reporting is currently not seen in BC or NS. When developing some of our indicators, ON already had an algorithm where the team discussed the merits of modifying or adapting to BC or NS. For example, the denominator for the ‘Diabetes Management’ indicator should include all people meeting the overall study criteria who have diabetes. Ontario was the only province that had a pre-existing Diabetes Database containing information on people who fulfill a validated algorithm for diabetes diagnosis. This algorithm identified diabetes cases based on physician claims, both diagnostic and service codes, and hospital admissions over a two-year period [69,70]. This pre-existing validated algorithm provides ON with a more accurate denominator. For many indicators, there was much discussion amongst the study team about how much precision was needed within the algorithm, whether ON’s previously developed algorithm would be comparable with measures derived from the other provinces, and whether a modified version was needed to achieve comparability with BC and NS. In this case, BC already had a similar pre-existing definition for identifying patients with diabetes but no separate database. Nova Scotia also had these data available but also did not have a separate database. Therefore, the same denominator identification algorithm was adopted by all three provinces, restricting to years of observation available for all three.

While the above represents a case where all provinces could agree upon and arrive at the same definition, in other cases discussion revealed that some pre-existing algorithms were meaningful for ON’s goals but conceptually different from what would be a meaningful indicator of performance in other provinces. For example, for indicators pertaining to continuity and usual provider ON would prefer to only use data where the patient had been rostered to a provider, but BC and NS do not roster; results would be skewed if ON only used rostered patients. In other words, if each province conceptualized an ideal performance indicator regardless of data availability, there may be discrepancies. Comparability in this situation is more about values and context than data quality or completeness.

Differences in data granularity — the level of available details in the dataset — were also challenges in establishing comparable algorithms. We needed to balance decreased precision by reducing the level of details available from one province to gain comparability with the level of details available in other provinces. For example, to define the indicator relating to osteoporosis screening, we used physician billings to identify bone mineral density testing. While all provinces could use fee codes, ON had more options available. To make a comparable indicator, ON aggregated several codes (those indicating baseline and follow-up and those specifying the body site being tested) because there were fewer options for billing codes in other provinces [71]. Similar procedures were performed on other indicator algorithms across the three provinces.

## Discussion

The number of indicators that could be developed using administrative data to understand PC performance across Canada is limited to technical quality of care, continuity of care, and health service utilization. While there may be other PC indicators available within one province, they are not useful for examining PC across Canada. Developing algorithms for performance indicators using administrative data across jurisdictions remains time intensive and completed by few in Canada. Work has been completed in areas of cancer care [72,73] and palliative care [74,75]. This is the first project creating algorithms to measure PC indicators across BC, ON, and NS using administrative data. Differences in resources for working with administrative data were most profound between ON and the other two provinces (NS and BC).

Using technology and a ‘living document’ that analysts and staff could maintain were key success factors for this work. Platforms that enable research team members to continuously edit the information and add / remove details to facilitate the discussion are essential. Our work showed that comparable algorithms and similar indicators across provinces using administrative data is possible and can become easier by building on work already completed [76,77]. The process of creating comparable performance indicators promoted mutual learning from pre-existing approaches in each province and also promoted updates to existing approaches based on other identified models.

While administrative data do not cover many important aspects of PC performance, there are advantages to using comparable administrative data indicators to assess elements of PC performance. These data are inexpensive because they are already collected. The process of achieving comparable algorithms may lead to some increases in measurement error, though it may be better to have more meaningful statistical comparisons at the cost of slightly reduced internal validity [78]. It is essential to address the tension between internal validity of the indicators within a province and external validity of the algorithms across provinces. The intuitive desire of having the exact same coding across provinces was challenging and required compromise to achieve. So, a context-tailored definition was developed for each jurisdiction with individuals knowledgeable about each health system and agreement on the final algorithms. We adapted existing algorithms and generated new ones to create a suite of algorithms that could be reasonably applied across jurisdictions. For comparisons to be possible, each provincial analyst agreed to: 1) discuss how to define comparable indicators, 2) calculate the indicators using their own provincial data, and 3) share results.

Primary care planning, resource allocation and improving quality both at individual practice level and at the healthcare system level require accurate measurement of PC performance [79]. Importantly, country-wide comparable indicators are essential for learning health systems [33] as they allow ongoing comparisons or assessments of performance. The scarcity of data to measure PC performance hampers decision-maker and clinicians’ abilities to strengthen it. Hopefully initiatives such as the Strategy for Patient Oriented Research Canadian Data Platform can contribute to across province/territory administrative data algorithm development for PC [80]. Additionally, increasing the use and improving the quality of electronic medical records and patient experience surveys can facilitate the availability of robust data to fill the measurement gap [81]. There is high potential of PC data and performance measurement to be advanced through these data sources, and through other platforms and activities. 

### Limitations

Our work was limited to using administrative data across three provinces, BC, ON, and NS. These data are limited to community-dwelling residents and what was possible to measure using the available data. It does not include members of the military or those living on-reserve as their health care services are captured in federal databases. Similarly, the work of many important non-physician providers such as nurse practitioners is not captured. Variable service fee codes, inconsistencies in physician billing practices, and different service definitions are known challenges for cross-provincial initiatives to measure PC performance using administrative data [31,32]. For example, one jurisdiction may provide an after-hours bonus starting at midnight, while another’s starts at 8 p.m. Moreover, contextual differences add challenges for inter-provincial comparisons, particularly as family physicians may provide the same services in a variety of settings from community-based clinics, to emergency rooms, to long-term care and hospital in-patient wards. For example, there may be differences in resulting records if one province compensates physicians for a certain service such as hypertension management when performed in a rural emergency department, and another only if that service is provided in an outpatient clinic. Hence, the extent to which ambulatory care records capture PC services received by any given patient will vary depending on 1) where they received services and 2) how their utilization pattern is recorded in their province. 

## Conclusion

Arriving at comparable administrative data definitions across provinces is essential to enhance the performance of PC. This task is challenging and time consuming. However, this study provides some foundational work towards establishing PC performance measurements in an inter-jurisdictional manner [11–13]. We established 21 indicators using a variety of administrative data sources. We highlighted both challenges and strengths of our approach, where there are differences in data structure and content and data pooling is not a solution. This work can be used as an approach to develop comparable algorithms of PC performance in health systems comprising of different jurisdictions.

## Acknowledgments

The opinions, results and conclusions reported in this paper are those of the authors and are independent from the funding sources. No endorsement by data stewards or funding sources is intended or should be inferred.

This research was funded by the Canadian Institutes of Health Research (grant number TTF-128265) and the Michael Smith Foundation for Health Research (grant number PT-CPH-00001-134).

In British Columbia, data was made accessible by Population Data BC. All inferences, opinions, and conclusions drawn in this article are those of the authors, and do not reflect the opinions or policies of the Data Steward(s).

This study was supported by ICES, which is funded by an annual grant from the Ontario Ministry of Health and Long-Term Care (MOHLTC). Parts of this material are based on data and information compiled and provided by MOHLTC, Canadian Cancer Organization and the Canadian Institute for Health Information. The analyses, conclusions, opinions and statements expressed herein are solely those of the authors and do not reflect those of the funding or data sources; no endorsement is intended or should be inferred. Parts of this material are based on data and/or information from the Canadian Drug Product Database and Data Extract, compiled and provided by Health Canada, and used by ICES with the permission of the Minister of Health Canada, 2017. https://www.canada.ca/en/health-canada/services/drugs-health-products/drug-products/drug-product-database.html. We thank IMS Brogan Inc. for use of their Drug Information Database.

The data (or portions of the data) used in this report were made available by Health Data Nova Scotia of Dalhousie University. Although this research is based on data obtained from the Nova Scotia Department of Health and Wellness, the observations and opinions expressed are those of the authors and do not represent those of either Health Data Nova Scotia or the Department of Health and Wellness.

## Ethics statement

Transforming CBPHC delivery through comprehensive performance measurement and reporting (TRANSFORMATION)”, was reviewed and approved by the Behavioural Research Ethics Boards in the University of British Columbia ref. no. H13-1237, Ottawa Health Science Network ref. no. 20140458-01H, Bruyère Continuing Care ref. no. M16-14-029, and the Nova Scotia Health Authority ref. no. 1017461.

## Abbreviations

BC	British Columbia
CIHI	Canadian Institute for Health Information
DAD	Discharge Abstract Database
ED	Emergency Department
ON	Ontario
NACRS	the National Ambulatory Care and Reporting System Metadata database
NS	Nova Scotia
PC	primary care
PCPOP	Primary Care Population cohort
P/T	provincial/territorial

## References

[1] Tu J V, Donovan LR, Lee DS, Wang JT, Austin PC, Alter DA, et al Effectiveness of public report cards for improving the quality of cardiac care: The EFFECT Study: a randomized trial. J Am Med Assoc. 2009 12 2;302(21):2330–7. DOI: 10.1001/jama.2009.1731 19923205

[2] The Commonwealth Fund. 2011 Commonwealth Fund International Health Policy Survey [Internet]. New York, NY: The Commonwealth Fund; 2011 [cited 2017 Dec 7]. Available from: http://www.commonwealthfund.org/interactives-and-data/surveys/international-health-policy-surveys/2011/2011-international-survey

[3] Smith MA, Wright A, Queram C, Lamb GC. Public reporting helped drive quality improvement in outpatient diabetes care among Wisconsin physician groups. Health Aff [Internet]. 2012;31:3):570–7. Available from: https://www.ncbi.nlm.nih.gov/pmc/articles/PMC3329125/ DOI: 10.1377/hlthaff.2011.0853 PMC3329125 22392668

[4] Faber M, Bosch M, Wollersheim H, Leatherman S, Grol R. Public reporting in health care: how do consumers use quality-of-care information? A systematic review. Med Care [Internet]. 2009;47(1):1–8. Available from: https://www.ncbi.nlm.nih.gov/pubmed/19106724 10.1097/mlr.0b013e3181808bb5 19106724

[5] Watson DE. For discussion: a roadmap for population-based information systems to enhance primary healthcare in Canada. Healthc Policy [Internet]. 2009 11 [cited 2013 Aug 23];5(Special Issue):105–20. Available from: http://www.pubmedcentral.nih.gov/articlerender.fcgi?artid=2906208&tool=pmcentrez&rendertype=abstract 10.12927/hcpol.2009.21190 PMC2906208 21037907

[6] Hibbard JH, Greene J, Sofaer S, Firminger K, Hirsh J. An experiment shows that a well-designed report on costs and quality can help consumers choose high-value health care. Health Aff [Internet]. 2012;31(3):560–8. Available from: https://www.ncbi.nlm.nih.gov/pubmed/22392666 DOI: 10.1377/hlthaff.2011.1168 22392666

[7] Powell AE, Davies HT, Thomson RG. Using routine comparative data to assess the quality of health care: understanding and avoiding common pitfalls. Qual Saf Heal Care. 2003;12(2):122–8. 10.1136/qhc.12.2.122 PMC1743685 12679509

[8] Grunfeld E, Lethbridge L, Dewar R, Lawson B, Paszat LF, Johnston G, et al Towards using administrative databases to measure population-based indicators of quality of end-of-life care: testing the methodology. Palliat Med [Internet]. 2006;20(8):769–77. Available from: http://pmj.sagepub.com/content/20/8/769.abstract 10.1177/0269216306072553 17148531 PMC3741158

[9] Ellins J, McIver S. Supporting patients to make informed choices in primary care: what works? [Internet]. Birmingham: University of Birmingham Health Services Management Centre; 2009 [cited 2017 Dec 7]. Available from: http://epapers.bham.ac.uk/747/

[10] Lavis JN, Permanand G, Oxman AD, Lewin S, Fretheim A. SUPPORT Tools for evidence-informed health Policymaking (STP) 13: Preparing and using policy briefs to support evidence-informed policymaking. Heal Res Policy Syst. 2009;7(Suppl 1):S13 10.1186/1478-4505-7-S1-S13 PMC3271824 20018103

[11] Lavis JN, Boyko JA, Oxman AD, Lewin S, Fretheim A. SUPPORT Tools for evidence-informed health Policymaking (STP) 14: Organising and using policy dialogues to support evidence-informed policymaking. Heal Res Policy Syst. 2009;7 Suppl 1:S14 10.1186/1478-4505-7-S1-S14 PMC3271825 20018104

[12] Van Walraven C, Dhalla IA, Bell C, Etchells E, Stiell IG, Zarnke K, et al Derivation and validation of an index to predict early death or unplanned readmission after discharge from hospital to the community. Can Med Assoc J [Internet]. 2010;182(6):551–7. Available from: http://www.cmaj.ca/content/182/6/551.abstract 10.1503/cmaj.091117 20194559 PMC2845681

[13] Macinko J, Starfield B, Shi L. The contribution of primary care systems to health outcomes within Organization for Economic Cooperation and Development (OECD) countries, 1970-1998. Heal Serv Res. 2003;38(3):831–65. 10.1111/1475-6773.00149 PMC1360919 12822915

[14] Starfield B, Shi L. Policy relevant determinants of health: an international perspective. Health Policy (New York). 2002;60(3):201–18. 10.1016/S0168-8510(01)00208-1 11965331

[15] Starfield B. Primary care: An increasingly important contributor to effectiveness, equity, and efficiency of health services. SESPAS report 2012. Gac Sanit [Internet]. 2012;26(Suppl. 1):20–6. Available from: http://dx.doi.org/10.1016/j.gaceta.2011.10.009 DOI: 10.1016/j.gaceta.2011.10.009 22265645

[16] Shortell SM, Casalino LP. Health care reform requires accountable care systems. JAMA. 2008;300(1):95–7. 10.1001/jama.300.1.95 18594045

[17] Berta W, Barnsley J, Brown A, Murray M. In the eyes of the beholder: Population perspectives on performance priorities for primary care in Canada. Healthc Policy. 2008;4(2):86–100. 10.12927/hcpol.2008.20170 19377373 PMC2645216

[18] Centers for Disease Control and Prevention. Ambulatory health care data Survey instruments: NAMCS survey instruments [Internet]. Vol. 2012 Atlanta, GA: Centers for Disease Control and Prevention; 2011 Available from: http://www.cdc.gov/nchs/ahcd/ahcd_survey_instruments.htm

[19] Canadian Institute for Health Information. Experiences with primary health care in Canada [Internet]. Ottawa, Ont.: Canadian Institute for Health Information; 2009 Available from: https://secure.cihi.ca/free_products/cse_phc_aib_en.pdf

[20] Glazier RH, Zagorski BM, Rayner J. Comparison of primary care models in Ontario Toronto, ON: Institute for Clinical Evaluative Sciences; 2012.

[21] Mangin D, Parascandalo J, Khudoyarova O, Agarwal G, Bismah V, Orr S. Multimorbidity, eHealth and implications for equity: a cross-sectional survey of patient perspectives on eHealth. BMJ Open [Internet]. 2019;9(2). Available from: http://bmjopen.bmj.com/DOI: 10.1136/bmjopen-2018-023731 PMC6377536 30760515

[22] Veillard J, Huynh T, Ardal S, Kadandale S, Klazinga NS, Brown AD. Making health system performance measurement useful to policy makers: Aligning strategies, measurement and local health system accountability in Ontario. Healthc Policy [Internet]. 2010 2 [cited 2015 Feb 19];5(3):49–65. Available from: http://www.pubmedcentral.nih.gov/articlerender.fcgi?artid=2831733&tool=pmcentrez&rendertype=abstract DOI: 10.12927/hcpol.2013.21639 21286268 PMC2831733

[23] Fung CH, Lim Y-W, Mattke S, Damberg C, Shekelle PG. Systematic review: The evidence that publishing patient care performance data improves quality of care. Ann Intern Med. 2008;148(2):111–23. 10.7326/0003-4819-148-2-200801150-00006 18195336

[24] Roland M. Linking physicians’ pay to the quality of care: A major experiment in the United Kingdom. N Engl J Med. 2004;351(14):1448–54. 10.1056/nejmhpr041294 15459308

[25] National Health Service D of H. The Quality and Outcomes Framework [Internet]. Vol. 2012 London, EN: Precedent; 2012 Available from: http://www.ic.nhs.uk/statistics-and-data-col lections/audits-and-performance/the-quality- and-outcomes-framework

[26] Health Quality Ontario. Quality Improvement [Internet]. 2017 [cited 2017 Oct 25]. Available from: http://www.hqontario.ca/Quality-Improvement

[27] BC Patient Safety & Quality Council. BC Patient Safety & Quality Council [Internet]. Vol. 2012 Vancouver, BC; 2008 Available from: http://www.bcpsqc.ca/contact.html

[28] Blendon RJ, Schoen C, DesRoches C, Osborn R, Zapert K. Common concerns amid diverse systems: Health care experiences in five countries. Health Aff [Internet]. 2003;22(3):106–21. Available from: http://content.healthaffairs.org/cgi/content/abstract/22/3/106 10.1377/hlthaff.22.3.106 12757276

[29] Blendon RJ, Schoen C, Donelan K, Osborn R, DesRoches CM, Scoles K, et al Physicians’ views on quality of care: A five-country comparison. Health Aff [Internet]. 2001;20(3):233–43. Available from: http://content.healthaffairs.org/cgi/content/abstract/20/3/233 10.1377/hlthaff.20.3.233 11585172

[30] Schoen C, Davis K, DesRoches C, Donelan K, Blendon R, Strumpf E. Equity in health care across five nations: Summary findings from an international health policy survey. The Commonwealth Fund. 2000. 11584833

[31] Schoen C, Osborn R, Huynh PT, Doty M, Zapert K, Peugh J, et al Taking the pulse of health care systems: Experiences of patients with health problems in six countries. Health Aff. 2005;W5:509–25. 10.1377/hlthaff.w5.509 16269444

[32] Schoen C, Osborn R, Trang Huynh P, Doty M, Davis K, Zapert K, et al Primary Care and Health System Performance: Adults’ Experiences in Five Countries. Health Aff. 2004;23(W4):487–503. 10.1377/hlthaff.w4.487 15513956

[33] Psek WA, Stametz RA, Bailey-Davis LD, Davis D, Darer J, Faucett WA, et al Operationalizing the learning health care system in an integrated delivery system. EGEMS (Washington, DC) [Internet]. 2015 [cited 2018 Dec 11];3(1):1122 Available from: http://www.ncbi.nlm.nih.gov/pubmed/25992388 DOI: 10.13063/2327-9214.1122 PMC4434917 25992388

[34] Morain SR, Kass NE, Grossmann C. What allows a health care system to become a learning health care system: Results from interviews with health system leaders. Learn Heal Syst [Internet]. 2017 1 1 [cited 2018 Dec 11];1(1):e10015 Available from: http://doi.wiley.com/10.1002/lrh2.10015 DOI: 10.1002/lrh2.10015 PMC6516720 31245552

[35] World Health Orgnization. Primary health care [Internet]. 2020 [cited 2020 Feb 20]. Available from: https://www.who.int/health-topics/primary-health-care#tab=tab_1

[36] Haggerty J, Burge F, Lévesque J-F, Gass D, Pineault R, Beaulieu M-D, et al Operational definitions of attributes of primary health care: consensus among Canadian experts. Ann Fam Med [Internet]. 2007 1 1 [cited 2015 Jan 29];5(4):336–44. Available from: http://www.ncbi.nlm.nih.gov/pubmed/17664500 DOI: 10.1370/afm.682 17664500 PMC1934980

[37] Hogg W, Rowan M, Russell G, Geneau R, Muldoon L. Framework for primary care organizations: The importance of a structural domain. Int J Qual Heal Care [Internet]. 2008;20(5):308–13. Available from: http://intqhc.oxfordjournals.org/content/20/5/308.abstract DOI: 10.1093/intqhc/mzm054 PMC2533520 18055502

[38] Wong ST, Haggerty JL. Measuring patient experiences in primary health care: A review and classification of items and scales used in publicly-available questionnaires UBC Centre for Health Services and Policy Research. Vancouver BC; 2013.

[39] Cadarette SM, Wong L. An Introduction to Health Care Administrative Data. Can J Hosp Pharm [Internet]. 2015;68(3):232–7. Available from: http://www.ncbi.nlm.nih.gov/pmc/articles/PMC4485511/ DOI: https://doi.org//10.4212%2Fcjhp.v68i3.1457 26157185 10.4212/cjhp.v68i3.1457 PMC4485511

[40] Health Quality Ontario. MyPractice primary care: A tailored report for quality of care [Internet]. 2017 Available from: http://www.hqontario.ca/Quality-Improvement/ Guides-Tools-and-Practice-Reports/Primary-Ca re

[41] The University of British Columbia. TRANSFORMATION: Measuring and improving the performance of primary health care in Canada [Internet]. [cited 2018 Sep 21]. Available from: http://www.transformationphc.ca/

[42] Marchildon GP. Health Systems in Transition - Canada, Second Edition. Health Systems in Transition. University of Toronto Press; 2013 1–186 p.

[43] Physicians in Canada, 2016: Summary Report [Internet]. 2017 [cited 2020 Apr 2]. Available from: www.cihi.cacopyright@cihi.ca ISBN978-1-77109-635-5

[44] Canadian Institute for Health Information (CIHI). National Ambulatory Care Reporting System Metadata (NACRS) | CIHI [Internet]. [cited 2018 Jul 9]. Available from: https://www.cihi.ca/en/national-ambulatory-c are-reporting-system-metadata

[45] Canadian Institute of Health Information. Discharge Abstract Database Metadata (DAD) | CIHI.

[46] British Columbia Ministry of Health [creator] (2011): Medical Services Plan (MSP) Payment Information File. V2. Population Data BC [publisher]. Data Extract. MOH (2011). http://www.popdata.bc.ca/data.

[47] BC Ministry of Health [creator] (2011): PharmaNet. V2. BC Ministry of Health [publisher]. Data Extract. Data Stewardship Committee (2011). http://www.popdata.bc.ca/data [Internet]. Available from: https://www.popdata.bc.ca/data

[48] British Columbia Ministry of Health [creator] (2011): Medical Services Plan (MSP) Practitioner File. V2. Population Data BC [publisher]. Data Extract. MOH (2011). http://www.popdata.bc.ca/data.

[49] BC Vital Statistics Agency [creator] (2011): Vital Statistics Deaths. V2. Population Data BC [publisher]. Data Extract BC Vital Statistics Agency (2011). http://www.popdata.bc.ca/data.

[50] Population Data BC | www.popdata.bc.ca [Internet]. [cited 2019 Apr 25]. Available from: https://www.popdata.bc.ca/

[51] Lavoie JG, Wong S, Katz A, Sinclair S. Opportunities and Barriers to Rural, Remote and First Nation Health Services Research in Canada: Comparing Access to Administrative Claims Data in Manitoba and British Columbia. Healthc Policy [Internet]. 2016 [cited 2019 Apr 25];12(1):52–8. Available from: http://www.ncbi.nlm.nih.gov/pubmed/27585026 PMC5008131 27585026

[52] Young GJ. Multistakeholder regional collaboratives have been key drivers of public reporting, but now face challenges. Health Aff [Internet]. 2012;31(3):578–84. Available from: https://www.ncbi.nlm.nih.gov/pubmed/22392669 DOI: 10.1377/hlthaff.2011.1201 22392669

[53] Dahrouge S, Hogg W, Younger J, Muggah E, Russell G, Glazier RH. Primary care physician panel size and quality of care: A population-based study in Ontario, Canada. Ann Fam Med [Internet]. 2016;14(1):26–33. Available from: https://www.scopus.com/inward/record.uri?eid=2-s2.0-84955087198&partnerID=40&md5=6fafaeb8566a0e24683d5c93d54b2a4d DOI: 10.1370/afm.1864 26755780 PMC4709152

[54] Hedden LK. Beyond full-time equivalents: Gender differences in activity and practice patterns for BC’s primary care physicians [Internet]. The University of British Columbia; 2015 Available from: http://hdl.handle.net/2429/55858 DOI: 10.14288/1.0221359

[55] Lavergne MR, Law MR, Peterson S, Garrison S, Hurley J, Cheng L, et al A population-based analysis of incentive payments to primary care physicians for the care of patients with complex disease. Can Med Assoc J [Internet]. 2016;188(15):1065–6. Available from: http://www.cmaj.ca/cgi/doi/10.1503/cmaj.160692 DOI: 10.1503/cmaj.160692 27527484 PMC5056888

[56] Dahrouge S, Seale E, Hogg W, Russell G, Younger J, Muggah E, et al A comprehensive assessment of family physician gender and quality of care: A cross-sectional analysis in Ontario, Canada. Med Care. 2016;54(3):277–86. DOI: 10.1097/MLR.0000000000000480 26765146

[57] Katz A, Valdivia J, Chateau D, Taylor C, Walld R, McCulloch S, et al A comparison of models of primary care delivery in Winnepeg [Internet]. Winnipeg, MB; 2016 [cited 2017 Jul 24]. Available from: http://mchp-appserv.cpe.umanitoba.ca/deliverablesList.html

[58] Lavergne MR, Peterson S, McKendry R, Sivananthan S, McGrail K. Full-service family practice in British Columbia: Policy interventions and trends in practice, 1991-2010. Healthc Policy. 2014;9(4):32–47. DOI: 10.1097/MLR.0000000000000310 PMC4749884 24973482

[59] Mcgrail K, Lavergne R, Lewis SJ, Peterson SLM, Barer M, Garrison SR. Classifying physician practice style: A new approach using administrative data in British Columbia. Med Care. 2015;53(3):276–82. DOI: 10.1097/MLR.0000000000000310 25634088

[60] Stukel TA, Croxford R, Rahman F, Bierman AS, Glazier RH. Variations in quality indicators across Ontario physician networks [Internet]. Toronto; 2016 [cited 2017 Dec 7]. Available from: https://www.ices.on.ca/Publications/Atlases-and-Reports/2016/Variations-in-Quality-Indicators-Across-Ontario-Physician-Networks

[61] Health Quality Ontario. Primary care practice report: Technical appendix [Internet]. 2016 Available from: http://www.hqontario.ca/Quality-Improvement/Guides-Tools-and-Practice-Reports/Primary-Care

[62] Jaakkimainen L, Klein-Geltink JE, Guttman A, Barnsley J, Zagorski BN, Kopp A, et al Indicators of primary care based on administrative data In: Jaakimanian L, Upshur R, Klein-Geltink J, Leong A, Maaten S, Schultz S, et al, editors. Primary care in Ontario: ICES atlas. Toronto: Institute for Clinical Evaluative Sciences; 2006 p. 207–50.

[63] Smith MJ, Liehr PR. Understanding Middle Range Theory by Moving Up and Down the Ladder of Abstraction In: Smith Patricia R. MJL, editor. New York: Springer Publishing Company; p. 15–32. Available from: https://connect.springerpub.com/content/book/978-0-8261-5992-2/part/part01/chapter/ch02 DOI: 10.1891/9780826159922.0002

[64] Etz RS, Gonzalez MM, Brooks EM, Stange KC. Less AND more are needed to assess primary care. J Am Board Fam Med [Internet]. 2017;30(1):13–5. Available from: http://www.ncbi.nlm.nih.gov/pubmed/28062812 DOI: 10.3122/jabfm.2017.01.160209 28062812

[65] Stange KC, Etz RS, Gullett H, Sweeney SA, Miller WL, Jaén CR, et al Metrics for assessing improvements in primary health care. Annu Rev Public Health [Internet]. 2014 1 [cited 2014 Jul 10];35(1):423–42. Available from: http://www.annualreviews.org/doi/abs/10.1146/annurev-publhealth-032013-182438 DOI: 10.1146/annurev-publhealth-032013-182438 24641561 PMC6360939

[66] Knowlton J, Belnap T, Patelesio B, Priest E, von Recklinghausen F, Taenzer AH. A Framework for Aligning Data from Multiple Institutions to Conduct Meaningful Analytics. eGEMs (Generating Evid Methods to Improv patient outcomes). 2017; DOI: 10.5334/egems.195 PMC5982973 29881753

[67] Qualls LG, Phillips TA, Hammill BG, Topping J, Louzao DM, Brown JS, et al Evaluating Foundational Data Quality in the National Patient-Centered Clinical Research Network (PCORnet(R)) EGEMS (Washington, DC) 2018 4;6(1):3 DOI: 10.5334/egems.199 PMC5983028 29881761

[68] Hogg WE, Tipper B. Development of a national framework for performance measurement in primary care. In: Primary Health Care Research Rounds. 2012.

[69] Creatore MI, Moineddin R, Booth G, Manuel DH, DesMeules M, McDermott S, et al Age- and sex-related prevalence of diabetes mellitus among immigrants to Ontario, Canada. CMAJ [Internet]. 2010 5 [cited 2019 Jun 13];182(8):781–9. Available from: http://www.ncbi.nlm.nih.gov/pubmed/20403889 DOI: 10.1503/cmaj.091551 PMC2871200 20403889

[70] Hux JE, Ivis F, Flintoft V, Bica A. Diabetes in Ontario: determination of prevalence and incidence using a validated administrative data algorithm. Diabetes Care [Internet]. 2002 3 1 [cited 2019 Jun 13];25(3):512–6. Available from: http://www.ncbi.nlm.nih.gov/pubmed/11874939 DOI: 10.2337/diacare.25.3.512 11874939

[71] Kueper JK, Alsabbagh MW, Peterson S, Wong ST. Achieving cross provincial comparisons of osteoporosis screening performance from administrative health data. Int J Popul Data Sci [Internet]. 2019 12 5;4(1):24 Available from: https://ijpds.org/article/view/1116 DOI: 10.23889/ijpds.v4i1.1116 PMC8144816 34095537

[72] Groome PA, McBride ML, Jiang L, Kendell C, Decker KM, Grunfeld E, et al Lessons Learned: It Takes a Village to Understand Inter-Sectoral Care Using Administrative Data across Jurisdictions. Int J Popul Data Sci [Internet]. 2018 11 12 [cited 2019 May 7];3(3). Available from: https://ijpds.org/article/view/440 DOI: 10.23889/ijpds.v3i3.440 PMC7299469 32935017

[73] Barbera L, Seow H, Sutradhar R, Chu A, Burge F, Fassbender K, et al Quality of end-of-life cancer care in Canada: a retrospective four-province study using administrative health care data. Curr Oncol [Internet]. 2015 10 [cited 2019 Jun 13];22(5):341–55. Available from: http://www.ncbi.nlm.nih.gov/pubmed/26628867 DOI: 10.3747/co.22.2636 26628867 PMC4608400

[74] Guthrie DM, Harman LE, Barbera L, Burge F, Lawson B, McGrail K, et al Quality Indicator Rates for Seriously Ill Home Care Clients: Analysis of Resident Assessment Instrument for Home Care Data in Six Canadian Provinces. J Palliat Med [Internet]. 2019 5 16 [cited 2019 Aug 19];jpm.2019.0022. Available from: http://www.ncbi.nlm.nih.gov/pubmed/31094608 DOI: 10.1089/jpm.2019.0022 31094608

[75] Urquhart R, Kotecha J, Kendell C, Martin M, Han H, Lawson B, et al Stakeholders’ views on identifying patients in primary care at risk of dying: a qualitative descriptive study using focus groups and interviews. Br J Gen Pract [Internet]. 2018 9 [cited 2019 Aug 19];68(674):e612–20. Available from: http://bjgp.org/lookup/doi/10.3399/bjgp18X698345 DOI: 10.3399/bjgp18X698345 30104331 PMC6104853

[76] Lawson B, Sampalli T, Warner G, Burge F, Moorhouse P, Gibson R, et al Improving Care for the Frail in Nova Scotia: An Implementation Evaluation of a Frailty Portal in Primary Care Practice. Int J Heal Policy Manag [Internet]. 2018 11 4 [cited 2019 Apr 25];8(2):112–23. Available from: http://www.ncbi.nlm.nih.gov/pubmed/30980624 DOI: 10.15171/ijhpm.2018.102 PMC6462204 30980624

[77] Warner G, Lawson B, Sampalli T, Burge F, Gibson R, Wood S. Applying the consolidated framework for implementation research to identify barriers affecting implementation of an online frailty tool into primary health care: a qualitative study. BMC Health Serv Res [Internet]. 2018 12 31 [cited 2019 Apr 25];18(1):395 Available from: http://www.ncbi.nlm.nih.gov/pubmed/29855306 DOI: 10.1186/s12913-018-3163-1 29855306 PMC5984376

[78] Voss EA, Ma Q, Ryan PB. The impact of standardizing the definition of visits on the consistency of multi-database observational health research. BMC Med Res Methodol [Internet]. 2015 12 8 [cited 2018 Aug 15];15(1):13 Available from: http://bmcmedresmethodol.biomedcentral.com/articles/10.1186/s12874-015-0001-6 DOI: 10.1186/s12874-015-0001-6 25887092 PMC4369827

[79] Haj-Ali W, Hutchison B. Establishing a primary care performance measurement framework for Ontario. Healthc Policy [Internet]. 2017;12(3):66–79. Available from: http://www.longwoods.com/content/25026 DOI: 10.12927/hcpol.2017.25026 28277205 PMC5344364

[80] SPOR Canadian Data Platform | www.popdata.bc.ca.

[81] Terry AL, Stewart M, Cejic S, Marshall JN, De Lusignan S, Chesworth BM, et al A basic model for assessing primary health care electronic medical record data quality. BMC Med Inform Decis Mak [Internet]. 2019 2 12 [cited 2020 Feb 20];19(1):30 Available from: https://bmcmedinformdecismak.biomedcentral.com/articles/10.1186/s12911-019-0740-0 DOI: 10.1186/s12911-019-0740-0 30755205 PMC6373085

[82] Katz A, Enns J, Wong S, Williamson T, Singer A, McGrail K, et al Challenges associated with cross-jurisdictionary analyses using administrative health data and primary care electronic medical records in Canada. Int J Popul Data Sci. 2018;3(3):1–9. 10.23889/ijpds.v3i3.437 PMC8142948 34095523

[83] BC Data Scout | www.popdata.bc.ca [Internet]. [cited 2019 Jul 29]. Available from: https://www.popdata.bc.ca/resources/BCDataScout

